# The Great Northern Appeal

**Published:** 1920-10-16

**Authors:** E. E. Cooper

**Affiliations:** Lord Mayor. The Mansion House, London, E.C. 4


*To the Editor of* THE HOSPITAL.

### The Great Northern Appeal.

*To the Editor of* THE HOSPITAL.

SIR,—A meeting is being arranged here on St. Luke's Day, October 18, at 3 P.M., on behalf of the Great Northern Central Hospital, Holloway, and, as the Lady Mayoress and I know and appreciate the immense value to the Metropolis particularly and the nation generally of the institution's indispensable work, I am going to preside, in the hope that I shall secure for the hospital the help which it deserves at this critical time.

If the hospital, which serves a large and densely populated area, with a million residents, is to continue without curtailing its work, it must have at least £8,000 before December 31, in addition to any income it can possibly expect to receive. It is to help in raising this sum that I am holding the meeting, when I shall be supported by Lord Northampton, Chairman of the hospital, and distinguished speakers, and I do hope that your readers will give me their support, and if they cannot come to the meeting send me a remittance, no matter how small, so that an efficient hospital service in North London may continue.—Yours faithfully,

E. E. COOPER, Lord Mayor.

The Mansion House, London, E.C. 4,
October 6, 1920.

